# Experimental data of lithium-ion battery and ultracapacitor under DST and UDDS profiles at room temperature

**DOI:** 10.1016/j.dib.2017.01.019

**Published:** 2017-02-06

**Authors:** Yujie Wang, Chang Liu, Rui Pan, Zonghai Chen

**Affiliations:** Department of Automation, University of Science and Technology of China, Hefei 230027, PR China

## Abstract

This article provides the dataset of both the LiFePO_4_ type lithium-ion battery (LIB) behavior and the Maxwell ultracapacitor behavior. The dynamic stress test (DST) condition and the urban dynamometer driving schedule (UDDS) condition were carried out to analyze the battery/ultracapacitor features. The datasets were achieved at room temperature, in August, 2016. The shared data contributes to clarify the behavior of the LIBs and ultracapacitors and can be used to predict the state-of-charge (SOC) of the LIBs and ultracapacitors, which is also shown in the article of “Modeling and state-of-charge prediction of lithium-ion battery and ultracapacitor hybrids with a co-estimator” (United States Advanced Battery Consortium, 1996) [1].

**Specifications Table**

**Table:** 

Subject area	*Energy*
More specific subject area	*Energy management, System modeling and state estimation*
Type of data	*Table*
How data was acquired	*Performance test experiments (Laboratory)*
Data format	*Raw and analyzed (processed)*
Experimental factors	*Room temperature*
Experimental features	*Experiments were carried out by the Neware BTS-8000 which is produced by the Shenzhen Neware Technology Co., LTD. The sampling time was 1 s.*
Data source location	*University of Science and Technology of China, Hefei, PR China*
Data accessibility	*Data is within this article*

**Value of the data**

• We show the data of the LIBs and ultracapacitors, which can be used for SOC prediction of the battery/ultracapacitor hybrids.
• The data can be used to analyze the performance of LIBs and ultracapacitors, as well as to identify the model parameters of the LIBs and ultracapacitors.
• The data under the DST and UDDS profiles can be used to analyze the dynamic behavior of the LIBs and ultracapacitors.

## 1 Data

The shared data describes the behavior of the LIBs (10 Ah) and ultracapacitors (3000 F) under the DST [1] and UDDS [2] profiles at room temperature in August, 2016 (in Appendix A.). Contents include the measured voltage, the load current (we define that the load current is negative when discharging, and positive when charging), and the sampling time. Those data can be used to analyze the dynamic behavior of the LIBs and ultracapacitors, and also can be used to predict the SOC or state-of-energy (SOE) [3].

## 2 Experimental design, materials and methods

In order to test the performance and dynamic behavior of the LIBs and ultracapacitors, the Neware BTS-8000 (produced by the Shenzhen Neware Technology Co., LTD.) is used to provide programmable DC power supply and electrical load functions for charging and discharging. A personal computer is used for data record and storage. The type of the LIB we tested is IFP-1665130-10 Ah (produced by Fujian Brother Electric CO., LTD of China) and the type of the ultracapacitor is BCAP3000-P270 2.7 V/3.0 Wh (produced by Maxwell Technologies, Inc.). The LIB pack is series connected by four batteries.

1) The LIB pack and ultracapacitor were discharged under DST current profile (Appendix A. mmc1).
2) The LIB pack and ultracapacitor were discharged under UDDS current profile (Appendix A. mmc2).
