# Supplementary material for: Experimental data of lithium-ion battery and ultracapacitor under DST and UDDS profiles at room temperature
Source: Data Brief. 2017 Feb 6;12:161–3. doi: 10.1016/j.dib.2017.01.019 (PMC5396853; doi:10.1016/j.dib.2017.01.019)
Supplement: Supplementary file 3 — Supplementary material [file mmc3.docx]

Dear editor(s) of DIB,

Attached, please find our manuscript entitled “Experimental data of lithium-ion battery and ultracapacitor under DST and UDDS profiles at room temperature”

This article provides the dataset of both the lithium-ion battery and the ultracapacitor behaviors. The dynamic stress test condition and the urban dynamometer driving schedule condition were carried out to analyze the battery/ultracapacitor features. The datasets were achieved at room temperature, in August, 2016. The shared data contributes to clarify the behavior of the LIBs and ultracapacitors and can be used to predict the state-of-charge of the LIBs and ultracapacitors.

The authors whose names are listed immediately below certify that they have NO affiliations with or involvement in any organization or entity with any financial interest (such as honoraria; educational grants; participation in speakers’ bureaus; membership, employment, consultancies, stock ownership, or other equity interest; and expert testimony or patent-licensing arrangements), or non-financial interest (such as personal or professional relationships, affiliations, knowledge or beliefs) in the subject matter or materials discussed in this manuscript.

We the undersigned declare that this manuscript is original, has not been published before and is not currently being considered for publication elsewhere.

We wish to confirm that there are no known conflicts of interest associated with this publication and there has been no significant financial support for this work that could have influenced its outcome.

We confirm that the manuscript has been read and approved by all named authors and that there are no other persons who satisfied the criteria for authorship but are not listed.

We further confirm that the order of authors listed in the manuscript has been approved by all of us.

We confirm that we have given due consideration to the protection of intellectual property associated with this work and that there are no impediments to publication, including the timing of publication, with respect to intellectual property. In so doing we confirm that we have followed the regulations of our institutions concerning intellectual property.

We understand that the Corresponding Author is the sole contact for the Editorial process (including Editorial Manager and direct communications with the office). He is responsible for communicating with the other authors about progress, submissions of revisions and final approval of proofs.

We confirm that we have provided a current, correct email address which is accessible by the Corresponding Author and which has been configured to accept email from Elsevier DIB department.

Yujie Wang, PhD candidate

Department of Automation, University of Science and Technology of China, Hefei, PR China.

**Signature:**

**Date:** September 19, 2016

Chang Liu, M.A

Department of Automation, University of Science and Technology of China, Hefei, PR China.

**Signature:**

**Date:** September 19, 2016

Rui Pan, M.A

Department of Automation, University of Science and Technology of China, Hefei, PR China.

**Signature:**

**Date:** September 19, 2016

Zonghai Chen, Professor

Department of Automation, University of Science and Technology of China, Hefei, PR China.

**Signature: *on behalf of Professor Chen***

**Date:** September 19, 2016

Corresponding Author: Professor Zonghai Chen, [chenzh@ustc.edu.cn](mailto:chenzh@ustc.edu.cn); Address: Department of Automation, University of Science and Technology of China, Hefei, 230027, PR China. Tel: +86 055163606104
